# India Rubber Hunting

**Published:** 1882-05

**Authors:** 


### INDIA RUBBER HUNTING.

The trees which yield the largest supply flourish along the banks of the Sinu and Aslalo rivers. The hunters, before entering the woods, provide themselves with guns, ammunition, flour, salt and tobacco. The flour is made from plantains, which are cut into slices, dried and ground, and is generally mixed with corn meal; this will keep sweet for months. For meat, the hunters depend upon the game they can kill. As soon as a rubb r tree is found he cleans a space round the trunk, cutting away all vines, underbrush, etc., and again marches on in search of more rubber trees, not returning to camp till night-fall. According to immemorial custom, a tree belongs to him who has cut round it. The hunt is continued until all the trees in the vicinity of the camp are thus secured, and then begins the work of gathering the rubber. A hole is dug in the ground near the rubber trees, unless another party is encamped near, in that case the holes are dug near the camp. The bark of the tree is first hacked with a "maché" as high as a man can reach, the cuts being in the form of a V, and the milk, or sap, collected as it exudes, and put into the hole which has been dug for it. After the sap ceases to flow from the cuts, a pile of wood or brush is made at the foot of the tree, and the tree itself is chopped down, the branches keeping one end of the tree off the ground, and the piles of wood at the foot of the tree doing the same at the other end, thus the tree is suspended. The hunter, after carefully placing large leaves on the ground under the tree, proceeds to cut gashes in the bark throughout its whole length. The sap is collected from the tree and from the leaves placed under it, and added to the milk first collected. The sap when it first exudes from the tree is as white as milk and as thick as cream, but it soon turns black on exposure to air and light if not properly watched and cared for. As soon as all the milk is placed in the hole, the rubber is coagulated by the addition of some substance, such as the root of "mechvacan," hard soap or other substances, and these cause the milk to coagulate so fast as to prevent escape of the water, which is always present in the fresh sap, and as the rubber and water will not mix, a piece of rubber coagulated in this manner is full of small cells containing water. It costs no more to make the rubber perfectly clear and transparent as amber, in which case it is infinitely more valuable, than to make it full of holes, water and dirt. As soon as all the rubber trees are cut down and the rubber coagulated, the pieces are strapped on the backs of the hunters by thongs of bark, and carried by them out to the bank of the river, and brought to market by canoe or raft.—*Land and Water.*

### CLOVES.

CLOVES grow on trees from twenty to thirty feet high, having a handsome pyramidal shape, with leaves that are large, glossy and evergreen. It is a native of Malacca, but is now grown in nearly all the spice islands of the Indian Ocean, the larger part of the crop coming from Amboyna, in the Island of Ternate. Many years ago the Dutch undertook to control the production of this spice, and to confine its growth to this island; they therefore destroyed the clove trees in the other spice islands, but the high prices which they demanded gradually led so its cultivation in territory outside of their jurisdiction, and they afterwards abandoned that policy. Still, most of the cloves now produced are grown in Dutch territory, and the high prices which have prevailed during the last year or two have been attributed partly to a failure in the crop at Ternate, and partly to the Acheen war, which considerably interfered with the supply usually derived from Sumatra.

The cloves of commerce are not, as many suppose, the fruit of the clove tree, but the flower buds. The ripe fruit in shape resembles a small olive, it is of a dark red color, with one or two cells containing as many seeds, and it is also aromatic to a certain extent and sometimes appears in commerce in a dried state under the curious name of "mother of cloves." It is not nearly so pungent, however, as the flower stems. Indeed, the whole tree—leaves, bark and wood—seems to be impregnated in some degree with the strong, distinctive clove flavor; the flower buds are the principal commercial product of the tree. When first gathered they are of a reddish color, but in the drying process, which is generally done by wood fires and partly in the sun, they turn a deep brown color, as they are when they reach us in America. Although the tree grows wild to some extent, it is regularly cultivated in plantations, the plants being set some ten or fifteen feet apart and carefully pruned and cared for.

